# Tagging single-nucleotide polymorphisms in candidate oncogenes and susceptibility to ovarian cancer

**DOI:** 10.1038/sj.bjc.6604947

**Published:** 2009-02-24

**Authors:** L Quaye, H Song, S J Ramus, A Gentry-Maharaj, E Høgdall, R A DiCioccio, V McGuire, A H Wu, D J Van Den Berg, M C Pike, E Wozniak, J A Doherty, M A Rossing, R B Ness, K B Moysich, C Høgdall, J Blaakaer, The Ovarian Cancer Association Consortium, D F Easton, B A J Ponder, I J Jacobs, U Menon, A S Whittemore, S Krüger-Kjaer, C L Pearce, P D P Pharoah, S A Gayther

**Affiliations:** 1 Gynaecological Oncology Department, UCL EGA Institute for Women's Health, University College London, London, UK; 2 Strangeways Research Laboratory, CR-UK Department of Oncology, University of Cambridge, Cambridge, UK; 3 Department of Virus, Hormones and Cancer, Institute of Cancer Epidemiology, Danish Cancer Society, Copenhagen, Denmark; 4 Department of Cancer Genetics, Roswell Park Cancer Institute, Buffalo, NY, USA; 5 Department of Health Research and Policy, Stanford University School of Medicine, Stanford, CA, USA; 6 Department of Preventive Medicine, University of Southern California, Keck School of Medicine, Los Angeles, CA, USA; 7 JD Program in Epidemiology, Division of Public Health Sciences, Fred Hutchinson Cancer Research Center, Seattle, WA, USA; 8 Department of Epidemiology and University of Pittsburgh Cancer Institute, Pittsburgh, PA, USA; 9 Gynaecology Clinic, The Juliane Marie Centre, Rigshospitalet, University of Copenhagen, Denmark, CR-UK; 10 Department of Gynaecology and Obstetrics, Aarhus University Hospital, Skejby, Aarhus, Denmark; 11 Department of Oncology, Strangeways Research Laboratory, Genetic Epidemiology Unit, University of Cambridge, Cambridge, UK

**Keywords:** risk of ovarian cancer, polymorphism, oncogene, association studies

## Abstract

Low–moderate risk alleles that are relatively common in the population may explain a significant proportion of the excess familial risk of ovarian cancer (OC) not attributed to highly penetrant genes. In this study, we evaluated the risks of OC associated with common germline variants in five oncogenes (*BRAF*, *ERBB2*, *KRAS*, *NMI* and *PIK3CA*) known to be involved in OC development. Thirty-four tagging SNPs in these genes were genotyped in ∼1800 invasive OC cases and 3000 controls from population-based studies in Denmark, the United Kingdom and the United States. We found no evidence of disease association for SNPs in *BRAF*, *KRAS*, *ERBB2* and *PIK3CA* when OC was considered as a single disease phenotype; but after stratification by histological subtype, we found borderline evidence of association for SNPs in *KRAS* and *BRAF* with mucinous OC and in *ERBB2* and *PIK3CA* with endometrioid OC. For *NMI*, we identified a SNP (rs11683487) that was associated with a decreased risk of OC (unadjusted *P*_dominant_=0.004). We then genotyped rs11683487 in another 1097 cases and 1792 controls from an additional three case–control studies from the United States. The combined odds ratio was 0.89 (95% confidence interval (CI): 0.80–0.99) and remained statistically significant (*P*_dominant_=0.032). We also identified two haplotypes in *ERBB2* associated with an increased OC risk (*P*_global_=0.034) and a haplotype in *BRAF* that had a protective effect (*P*_global_=0.005). In conclusion, these data provide borderline evidence of association for common allelic variation in the *NMI* with risk of epithelial OC.

Globally, ovarian cancer (OC) is responsible for approximately 125 000 deaths each year (Parkin *et al*, 2005). The risk to first-degree relatives of an OC case is increased above population rates (Stratton *et al*, 1997) and twin studies suggest that genes are more important than shared environment (Lichtenstein *et al*, 2000); however, most cases occur without any obvious evidence of inherited susceptibility (i.e., they appear to be sporadic). The two highly penetrant susceptibility genes *BRCA1* and *BRCA2* are responsible for approximately half of all families containing two or more ovarian cancer cases (Ford *et al*, 1998; Gayther *et al*, 1999; Ramus *et al*, 2007), but these genes account for less than 40% of the familial excess risk of OC (Antoniou and Easton, 2006). The remaining familial risks are thought to be due to combinations of multiple alleles that confer moderate- or low-penetrance susceptibility (Pharoah *et al*, 2002).

The most widely used approach to identify moderate/low-risk susceptibility alleles for cancer has been to study candidate genes in biologically relevant pathways. Proto-oncogenes are essential for normal cell function, particularly in the regulation of cell division, proliferation, survival, motility and apoptosis. Activating mutations or amplification of these genes generally elevates growth factor production and stimulates cell mitosis leading to neoplastic transformation (Rhim, 1988; Hogdall *et al*, 2003a). To date, few studies have reported on the OC risks associated with germline genetic variation in proto-oncogenes. A variant of borderline significance has been reported in the putative oncogene *AURKA* (earlier known as *STK15*) (Dicioccio *et al*, 2004). However, this was not confirmed in a larger consortium study (Ramus *et al*, 2008). Most of the oncogenes known to be altered in OC development have not yet been studied.

Of the oncogenes known to be involved in OC, *KRAS* is the most frequently mutated (Forbes *et al*, 2006). *KRAS* functions in the receptor tyrosine kinase pathway (Gemignani *et al*, 2003) and several other genes that function in this pathway are mutated in multiple tumour types (Cuatrecasas *et al*, 1997). Activating mutations of *KRAS* appear to be an early event in OC development, but predominantly tumours of the mucinous histological subtype (Gemignani *et al*, 2003). Mutations in codons 12 and 13 have been detected in approximately 50% of mucinous OCs (Gemignani *et al*, 2003). *BRAF,* in the mitogen-activated protein kinase pathway, is a downstream effector of *KRAS* and is critical in the transduction of cell growth signals (Cuatrecasas *et al*, 1998). Overexpression of *BRAF* has been found in a variety of cancers, and mutations have been reported in 12% of OCs (Gemignani *et al*, 2003; Russell and McCluggage, 2004; Sieben *et al*, 2004).

PIK3CA is the catalytic subunit of the lipid kinase phosphatidylinositol 3-kinase (PIK), which is involved in the regulation of cell proliferation, adhesion transformation, survival, apoptosis and motility (Volinia *et al*, 1994; Fruman *et al*, 1998; Cantley, 2002). The helical and kinase domains of *PIK3CA* are hotspots for mutations, which have been found in multiple tumour types including ovary, breast, lung, brain, colon and stomach (Muller *et al*, 2007). *PIK3CA* mutations have been shown to correlate with increased gene expression in several OC cells lines. Detectable amplification of the gene has also been shown in 58% of ovarian tumours using fluorescence *in situ* hybridisation (Shayesteh *et al*, 1999).

The human epidermal growth factor receptor-2 gene, *ERBB2* (*HER-2*/*Neu*), is a transmembrane protein that acts as a growth factor receptor and is involved in cell proliferation and cell differentiation (Wu *et al*, 2004). Breast, prostate, lung, gastrointestinal, kidney, liver and bladder cancers have all shown an elevated expression of *ERBB2* (Wu *et al*, 2004). For OC, 20–30% of primary stage III/IV tumours show *ERBB2* overexpression (Hellstrom *et al*, 2001). Protein expression using antibody staining on a subset of ovarian tumours from the MALOVA study showed that 39% of the carcinomas overexpressed *ERBB2* (Hogdall *et al*, 2003b).

The *MYC* family of proto-oncogenes, including *NMYC* and *MYC*, and their interacting partners, are transcription factors that have a well-documented role in tumourigenesis. *MYC* overexpression caused by gene amplification induces uncontrolled hyper-proliferation and occurs in ∼35% of epithelial OCs. Another gene, the *NMYC* and *STAT* interactor (*NMI*), which interacts with *NMYC*, *MYC*, *MAX*, *FOS*, other transcription factors (Zhu *et al*, 1999) and *BRCA1* (Li *et al*, 2002), is overexpressed in human leukaemias and other cancers (Bao and Zervos, 1996).

The aim of this study was to evaluate the risks of OC associated with common genetic variation in five of the candidate oncogenes described above – *BRAF*, *ERBB*, *KRAS*, *NMI* and *PIK3CA* – using a SNP-tagging approach. To do this, we genotyped 34 common tagging SNPs (tSNPs) in 1816 invasive epithelial OC cases and 3000 unaffected controls from five different case–control studies from the United States, United Kingdom and Denmark as part of a multi-centre collaboration. We then evaluated one positive finding in a further 1097 cases and 1712 controls from three other US studies.

## Materials and methods

### Study individuals

In the first stage of this study, we genotyped OC cases and controls from five different populations. These were (1) The Danish MALOVA study (446 cases and 1221 controls); (2) The UK SEARCH study (730 cases and 855 controls); (3) The Genetic Epidemiology of Ovarian Cancer Study (GEOCS; previously FROC) from Stanford, CA, USA (327 cases and 429 controls); (4) The USC (A) study from Los Angeles, CA, USA (197 cases and 224 controls); and (5) the UKOPS study from the United Kingdom (116 cases and 271 controls). In stage 2, a putative positive association was followed up in three other case–control studies: (1) The USC (B) study, CA, USA (237 cases and 360 controls); (2) The DOVE study, Seattle, WA, USA (584 cases and 716 controls); and (3) The HOPE study, Pittsburgh, USA (276 cases and 636 controls). USC (A) and (B) are subsets of the same USC population. The USC (A) samples were collected between 2000 and 2004 and USC (B) samples were collected from 1993 to 1999. All study individuals were non-Hispanic Whites. Details for several of these studies have been published before (Dicioccio *et al*, 2004; Pearce *et al*, 2005; Song *et al*, 2006, 2007; Gayther *et al*, 2007; Rossing *et al*, 2007) and are summarised in Table 1. Local ethics committee approval was given for the collections and genotyping in all individuals.

### Candidate gene and tSNP selection

We chose to analyse candidate oncogenes for which there is evidence that the genes were amplified or mutated in OCs. The genes we chose to examine initially were *BCL2*, *BRAF*, *MYC*, *CTNNB1*, *EGFR*, *ERBB2*, *FGF3*, *HRAS*, *KIT*, *MDM2*, *NMI* and *PIK3CA*. Some of these genes were excluded if no HapMap genotyping data were available, if the gene was poorly tagged or if there were <3 tSNPs or >15 tSNPs in the genes. We used data from the CEPH population, from The International HapMap Project Data Rel 20/phase II Jan06 (www.hapmap.org), Haploview version 3.32 (Barrett *et al*, 2005) and Tagger (de Bakker *et al*, 2005) to select tSNPs that capture common genetic variation in each candidate gene, and putative regulatory regions up and downstream of the gene (within 5 kb), with a minimum squared correlation of 0.8 (*r*^2^⩾0.8). The multi-marker (aggressive) tagging option of Tagger was used to select tSNPs. If a selected tSNP failed assay design or genotyping, an alternative tagging SNP was chosen.

### Genotyping SNPs

A combination of iPLEX (Sequenom Inc., Hamburg, Germany) and TaqMan ABI 7900HT Sequence Detection System (Applied Biosystems, Warrington, UK) was used to genotype the samples as described earlier (Zhao *et al*, 2006). The MALOVA and SEARCH samples were genotyped by a combination of TaqMan and iPLEX; UKOPS, USC and GEOCS were genotyped with TaqMan only. Genotyping with iPLEX was performed at the Sequenom laboratory in Hamburg, Germany. TaqMan genotyping of stage 1 samples was performed at the Gynaecological Cancer Research Laboratories, University College London, and Strangeways Research Laboratory, University of Cambridge (both UK). For stage 2, samples were genotyped by TaqMan at the Keck School of Medicine, University of Southern California, USA. Genotyping was repeated for studies/plates when call rates were below 90%, if there were discordant duplicate samples or if negative controls tested positive (Song *et al*, 2006, 2007; Gayther *et al*, 2007).

### Statistical methods

Deviation from Hardy–Weinberg equilibrium (HWE) was assessed in controls within study populations using the standard *χ*^2^ test. Unconditional logistic regression was used to assess the relationship between each tSNP and risk of OC for each study and pooled across studies (stratified by study), with the primary test of association being a test for trend (*P*_trend_). The per-allele odds ratio and odds ratios for the heterozygote and rare homozygote relative to the common homozygote were estimated by stratified logistic regression. The programme TagSNPs (Stram *et al*, 2003) was used to model the relevant multi-marker haplotypes resulting from aggressive SNP tagging. Heterogeneity between study strata was tested by comparing logistic regression models with and without a genotype–stratum interaction term using the likelihood ratio test. All the reported *P*-values are two sided. There was no association in controls between age and genotype frequency for any of the SNPs, and adjusting for age did not materially alter the effect estimates and thus age was not included in the models (data not shown). Where there was evidence for association, we compared the fit of log-additive co-dominant, dominant and recessive genetic models using likelihood ratio tests.

We also conducted analyses to determine if haplotype effects were present. Haplotype blocks (regions of strong linkage disequilibrium) were defined using the confidence interval option of Haploview (Gabriel *et al*, 2002), with minor adjustments to include adjacent SNPs, but maintaining the cumulative frequency of the common haplotypes to >90%. All genes had one haplotype block, except *KRAS,* which had two blocks. Haplotype analysis was conducted using the programme TagSNPs (Stram *et al*, 2003). TagSNPs implements an expectation–substitution approach to account for the uncertainty caused by the unphased genotype data (Stram *et al*, 2003). The genotype data for GEOCS, MALOVA, SEARCH, UKOPS and USC (A) samples were used in this analysis. Unconditional logistic regression was used to test the association between each haplotype relative to the most common haplotype (Zaykin *et al*, 2002; Stram *et al*, 2003). Haplotypes that occurred with a frequency of 2% or greater in the combined data were considered ‘common’, and those with less than 2% frequency were pooled together as rare haplotypes.

## Results

Forty SNPs were selected to tag the common genetic variation in *BRAF*, *ERBB2*, *KRAS*, *NMI* and *PIK3CA*. Six of these (*PIK3CA*: rs1607237, rs6443624 and rs3729692; *KRAS*: rs11047912 and rs17388893; *BRAF*: rs11771946) failed assay design, manufacture or genotype testing and could not be efficiently tagged by any other SNP. Therefore, we were able to genotype 34 SNPs in total (Table 2). The tSNPs were selected using HapMap Data Rel 20/phase II on NCBI B35 assembly, dbSNP b125. For all five genes, we captured 176 of 188 (94%) common SNPs with *r*^2^>0.8. Tagging efficiencies were the same using the most recent HapMap data release (HapMap Data Rel 21a/phase II on NCBI B35 assembly dbSNPb125), which captured 199 of 212 (94%) of the common SNPs with *r*^2^>0.8.

This panel of SNPs was genotyped in five different OC case–control studies from the United Kingdom (SEARCH and UKOPS), United States (GEOCS and USC (A)) and Denmark (MALOVA). Combined, these studies comprise 1816 invasive epithelial OC cases and 3000 unaffected female controls. For most tSNPs, genotype distributions in controls were consistent with HWE in all populations in which genotyping passed quality control criteria. For one SNP (rs2699905), controls from GEOCS, SEARCH and UKOPS deviated significantly from HWE (*P*<0.01). Seven tSNPs (rs2952155, rs11047917, rs11551174, rs10487888, rs2865084, rs1733832 and rs289831) could not be genotyped for GEOCS, UKOPS and USC (A) because Taqman assays for these tSNPs failed assay manufacture. This is reflected in the variable numbers of cases and/or controls that were successfully genotyped for each tSNP, listed in Table 3.

### Association between genotype frequencies and OC risk

We found no evidence of association between tSNPs or multi-marker haplotypes in *BRAF*, *ERBB2*, *KRAS* and *PIK3CA* and susceptibility to invasive epithelial OC (Table 3 and Supplementary Table 1). A SNP in *NMI* (rs11683487) showed evidence of association with reduced risk of OC (heterozygous odds ratio (OR) 0.80 (95% confidence interval (CI) 0.69–0.93); homozygous OR 0.87 (95% CI 0.71–1.02); *P*_trend_=0.038) (Table 3). The best-fitting genetic model for this SNP was a dominant model (*P*=0.004) (rare allele carriers *vs* common allele homozygotes OR 0.81 (95% CI 0.71–0.94)). There was no statistically significant heterogeneity across studies for any SNP.

**Figure 1:**
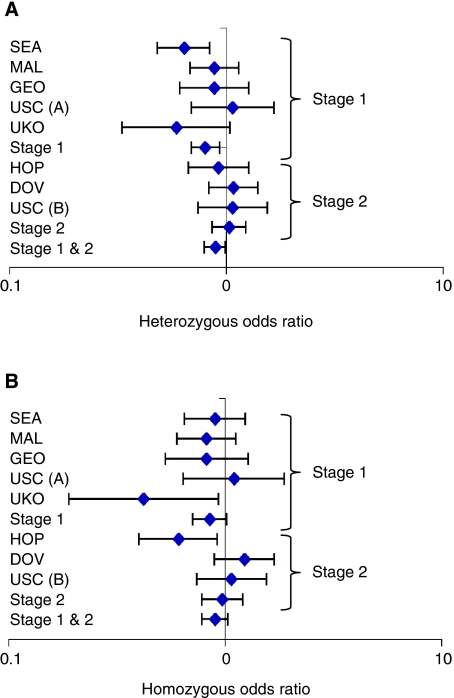
Forest plots of tSNP rs11683487 in the *NMI* gene in ovarian cancer case–control populations. SEA – SEARCH, UK; MAL – MALOVA, Denmark; GEO – GEOCS, USA; USC – University of Southern California, USA; DOV – DOVE, USA; HOP – HOPE, USA; UKO – UKOPS, UK. (**A**) Heterozygous and (**B**) homozygous odds ratios for all invasive ovarian cancer cases. All cases combined: nominal model – HetOR=0.89 (95% CI 0.79–0.99), HomOR 0.9 (0.78–1.03), *P*_trend_: 0.0831; dominant model – OR=0.87 (0.8–0.99), *P*_dominant_: 0.0317.

The association with rs11683487 was investigated further by performing a second stage of genotyping in three additional populations from the United States (USC (B); DOVE and HOPE) (Table 1). Together, these three studies comprised an additional 1097 cases and 1712 controls. There was no association between rs11683487 and the risk of OC in the samples used for validation on their own (*P*_dominant_=0.92; OR=1.01 (0.85–1.20)). After combining the data from both stages, the association with rs11683487 was weaker, but still statistically significant (*P*_dominant_=0.032; OR=0.89 (0.80–0.99); Figure 1A; Supplementary Table 2).

Earlier studies have shown that different histological subtypes of OC have different genetic and biological backgrounds and are associated with different aetiological pathways. Therefore, we stratified cases by histological subtype and repeated the analyses. In the combined sample set, there were 859 OC cases of the serous histological subtype, 274 endometrioid cases, 192 mucinous cases and 138 clear-cell cases. We found no additional evidence of genetic associations in the serous subtype, but we did find borderline evidence of association for one SNP each in the *ERBB2* and *PIK3CA* genes with endometrioid OC and for three SNPS each in the *BRAF* and *KRAS* genes and one SNP in the *NMI* gene, all associated with mucinous OC. These data are summarised in Table 4.

We performed tests of association with common haplotypes for the five genes. There was no evidence of association with OC risk for haplotypes in *KRAS* and *PIK3CA* (Table 5). We found statistically significant haplotype effects for *BRAF*, *ERBB2* and *NMI*. Two haplotypes from *ERBB2* were associated with an increased OC risk, h233 (OR=1.17 (1.02–1.34), *P*=0.022) and h411 (OR=1.19 (1.03–1.37), *P*=0.016), respectively. A haplotype in *BRAF*, h333423241, was associated with a decrease in the risk of OC (OR=0.81 (0.68–0.95), *P*=0.012). Global tests of association were significant for *BRAF* (*P*=0.005) and *ERBB2* (*P*=0.034). The association observed with the *NMI* haplotype was fully explained by the single tSNP association.

## Discussion

Somatic alterations that activate proto-oncogenes and drive cells towards unregulated proliferation are a well-documented feature of all cancers. It has also become clear that different combinations of oncogenes contribute to the development of different tumour types. *BRAF*, *ERBB2*, *KRAS* and *PIK3CA* are all oncogenes shown to be involved in OC development, and NMI interacts with the oncogenes *NMYC*, *MYC*, *MAX* and *FOS*. NMI has also been shown to form a complex with MYC and BRCA1 and therefore may play a role in breast cancer and OC (Li *et al*, 2002). In this study, we evaluated the association between 34 tSNPs in these genes and the risk of invasive epithelial OC using a case–control study design. To our knowledge, none of these genes has been investigated before for their association with invasive OC.

We found borderline evidence for a statistically significant association with disease risk for a tSNP, rs11683487, in intron 1 of the *NMI* gene. The common allele (G) occurs in the Caucasian population with a frequency of approximately 58% and we observed that the rare allele (T) was associated with a decreased risk of OC. The association for rs11683487 may be a false positive. Where the prior probability of association is low, very stringent significance levels are required to ensure that a detected association is true positive. Genome-wide significance is generally considered to be *P*<10^−7^ (Thomas *et al*, 2005). False-positive associations due to population stratification is also possible, but this seems an unlikely explanation for data from multiple studies from different populations in which the analyses were restricted to white subjects.

If the association we identified in *NMI* is real, then this could be either due to a direct causal effect of the tSNP or because the SNP is in linkage disequilibrium with the true causal variant, possibly in a different gene. *NMI* enhances the transcription of several other genes altered in OCs (*MYC*; *N-MYC*) when it is induced by interleukin-2 and interferon-*γ*. The role of *MYC* amplification in ovarian and other cancers is well established. A detailed mapping of SNPs at a locus on chromosome 8q, near *MYC*, has recently provided substantial evidence that this locus is associated with susceptibility to breast, prostate and ovarian cancer (Ghoussaini *et al*, 2008). A further link comes from the finding that NMI forms a complex with MYC and BRCA1 (Li *et al*, 2002).

The *NMI* SNP rs11683487 tags eight other SNPs with *r*^2^>0.8, one of which is a non-synonymous coding SNP (rs1048135) tagged with an *r*^2^=1, and the rare (G) allele codes for leucine instead of serine. We examined whether there is any evidence supporting the role of these SNPs in abrogating NMI function. The programme PMut (Ferrer-Costa *et al*, 2005) predicted that the rare allele (coding for leucine), with a score of 3/10 had a ‘pathological significance’ and was classed as ‘damaging’ using the SIFT programme (Cheng *et al*, 2006). The bioinformatics tool, PupaSNP (http://pupasuite.bioinfo.cipf.es/) (Conde *et al*, 2006; Reumers *et al*, 2008), also suggested that this allele may disrupt the binding of exonic splicing enhancers. In addition, PupaSNP indicated that rs11683487 and rs11730 may have transcription and translation regulatory functions, and that rs11730 may affect exon splicing.

We found no evidence of association with disease risk for polymorphisms in *BRAF*, *ERBB2*, *KRAS* and *PIK3CA* at *P*⩽0.05, when OC was considered as a single disease phenotype. The combined sample size from five studies provides 98% power at the 5% significance level to detect a co-dominant allele with a frequency of 0.3 that confers a relative risk of 1.2, and 95% power to detect a dominant allele with a frequency of 0.1 that confers a relative risk of 1.3. It is therefore unlikely that the common tagged variants in these genes contribute significantly to OC risk. However, we cannot rule out the possibility that associations exist for the known poorly tagged variants. With the most recent HapMap data (release 21a), of the 212 common variants, 199 were tagged with *r*^2^>0.8 and 205 with *r*^2^>0.5. Furthermore, even though tSNPs based on HapMap data are likely to tag most of the common SNPs, there is a possibility that other unknown common variants were poorly tagged, or that less common variants in these genes that influence disease susceptibility exist.

We must also consider the possibility that common variants within these genes confer susceptibility to specific subtypes of OC. There is evidence in the literature that the genetic changes associated with OC development differ for different histological subtypes (reviewed in Elmasry and Gayther, 2005). For example, somatic activating *KRAS* mutations are found to some extent in most OC subtypes, but are much more common in mucinous ovarian tumours. Also, germline *BRCA1* and *BRCA2* mutations tend to predispose to serous OCs (Lakhani *et al*, 2004). We found some evidence of association with disease risk for different histological subtypes, for SNPs in all five of the oncogenes studied, and it is perhaps interesting that SNPs in the *KRAS* gene and its downstream effector *BRAF* were associated with mucinous OC. However, the sample sizes after subtype stratification meant that these studies had insufficient power to detect associations at stringent levels of statistical significance, and so the data must be treated with caution. Much larger sample sizes, gathered through the ovarian cancer association consortium (OCAC), will be needed to establish if any of these associations are real.

Haplotype analysis identified significant associations in *BRAF*, *ERBB2* and *NMI*. The association between the *NMI* haplotype and risk of OC is explained by the single SNP rs11683487. The global test of haplotype effect was statistically significant for *BRAF* and *ERBB2*. Interestingly, the two haplotypes in *ERBB2*, which are significantly associated with increased risk of OC, contain the opposite allele at each SNP loci. Using HapMap data we evaluated whether these two putative risk haplotypes in *ERBB2* shared an untagged common variant, but this was not the case. It is possible that there is an as-yet-unidentified variant that tags both haplotypes. There may be an allele that is found only in the protective haplotype of *BRAF*, which was not captured with our genotyping.

This study is one of the several in the published literature to use the multi-centre OCAC to follow-up on putative susceptibility alleles for OC (e.g., Gayther *et al*, 2007; Pearce *et al*, 2008; Ramus *et al*, 2008). These studies highlight the importance of consortia for validating suggested genetic associations from case–control studies and for identifying novel susceptibility loci for the disease. In addition to dramatically increasing the power of association studies, another role of consortia like the OCAC, has been to implement stringent data quality and genotyping guidelines, which are likely to minimise reports of false-positive associations.

In conclusion, we genotyped 34 tSNPs that tag the common variants in *BRAF*, *ERBB2*, *KRAS*, *NMI* and *PIK3CA* in OC cases and controls. We found borderline evidence of a statistically significant association with invasive OC, for a SNP in *NMI* and haplotypes in *BRAF* and *ERBB2*. Further studies will be needed to confirm if this genetic risk association is real or not.

## Figures and Tables

**Table 1:** Ovarian cancer case–control populations included in this study

	**Controls**				**Cases**			
Population*	**Total**	**Age**	**Participation rate (%)**	**Ascertainment**	**Total**	**Age**	**Participation rate (%)**	**Ascertainment**
MALOVA (Denmark)	1221	35–79	67	Random selection of females from the computerised Central Population Register.	446	35–79	79	Incident cases diagnosed between 1994 and 1999 from municipalities of Copenhagen and Frederiksberg and surrounding counties.
SEARCH (UK)	855	39–77	84	Selected from the EPIC-Norfolk cohort of 25 000 individuals based in the same geographical regions as the cases.	730	21–74	69	Cases from East Anglian West Midlands and Trent regions of England. Prevalent cases diagnosed between 1991 and 1998; incident cases diagnosed 1998 onwards.
GEOCS (USA)	429	19–66	75	Random-digit dial identification from study area. Frequency matched to cases for race/ethnicity and 5-year age group.	327	23–64	75	Consecutive cases diagnosed from 1997 to 2002 in Greater Bay Area Cancer Registry San Francisco.
USC (A) (USA)	224	21–78	73	Neighbourhood recruited controls, frequency matched to cases for age and ethnicity from 2000 to 2004.	197	18–84	73	Rapid case ascertainment through Los Angeles Cancer Surveillance programme from 2000 to 2004.
UKOPS (UK)	271	50–76	97	Apparently healthy postmenopausal women from the general population participating in the United Kingdom Collaborative Trial of Ovarian Cancer Screening (UKCTOCS). All women followed up for cancers through the Office of National Statistics.	116	35–86	86	Incident cases from 10 gynaecological oncology National Health Service centres throughout the United Kingdom, from January 2006 onwards.
Total (stage 1)	3000				1816			
USC (B) (USA)	360	21–78	73	Controls recruited from same neighbourhoods as cases from 1993 to 1999, frequency matched to cases for age and ethnicity.	237	18–84	73	Cases recruited through the Los Angeles Cancer Surveillance programme from 1993 to 1999.
DOVE (USA)	716	35–74	82	Random-digit dial identification from study area. Frequency matched to cases for race/ethnicity and 5-year age group.	584	35–74	75	Cases diagnosed with primary invasive ovarian cancer between 2002 and 2005 from a 13-county area of Western Washington state.
HOPE (USA)	636	25–80	81	Identified in same regions as cases. Frequency matched for age and ethnicity. All participants undergo home interviews.	276	25–80	69	Variable source including physician offices cancer registries and pathology databases from counties of Western PA Eastern OH and Western NY.
Total (stage 2)	1792				1097			
TOTAL (stages 1 and 2)	**4712**				**2913**			

^*^All samples are population based. All individuals are non-hispanic Whites.

**Table 2:** tSNPs identified after genotyping in ovarian cancer cases and controls

**HapMap data**	**Gene**	**All SNPs**	**Criteria SNPs**	**Tagging SNPs**	**SNPs genotyped**	**SNPs captured *r*^2^>0.8**	**SNPs captured *r*^2^>0.8 (%)**	**SNPs captured *r*^2^>0.5**
Release 20^a^	*BRAF*	158	75	9	8	72/75	96	72/75
	*ERBB2*	16	6	3	3	6/6	100	6/6
	*KRAS*	59	46	11	9	42/46	91	44/46
	*NMI*	45	25	6	6	25/25	100	25/25
	*PIK3CA*	53	36	11	8	33/36	92	34/36
Total		**331**	**188**	**40**	**34**	**176/188**	**94**	**181/188**
Release 21a^b^	*BRAF*	164	75	9	8	72/75	96	72/75
	*ERBB2*	26	11	5	3	9/11	82	9/11
	*KRAS*	69	55	11	9	50/55	91	53/55
	*NMI*	52	32	6	6	32/32	100	32/32
	*PIK3CA*	60	39	11	8	36/39	92	39/39
Total		**371**	**212**	**42**	**34**	**199/212**	**94**	**205/212**

SNP=single-nucleotide polymorphism.

Criteria SNPs: minor allele frequency⩾0.05; Hardy–Weinberg equilibrium⩾0.01. The captured SNPs refer to the number and proportion of criteria SNPs covered by the HapMap release.

a The tagging SNPs genotyped were selected using HapMap Data Release 20.

b The genes were retagged using the new HapMap data release (21a), and the proportion of SNPs that were captured by the SNPs genotyped was determined.

**Table 3:** Genotype-specific risks (OR and 95% CI) of pooled stage 1 data

**Gene**	**SNP name**	**Case**	**Control**	**MAF**	**HetOR^a^ (95% CI)**	**HomOR^a^ (95% CI)**	***P*_trend_**
*BRAF*	rs10487888^b^	1680	2694	0.47	1.09 (0.93–1.28)	1.02 (0.86–1.22)	0.9
	rs1733832^b^^,^^c^^,^^d^	1159	2043	0.06	1.08 (0.86–1.36)	3.39 (0.96–11.89)	0.2
	rs1267622	1751	2880	0.24	0.99 (0.87–1.12)	0.97 (0.75–1.26)	0.79
	rs13241719	1602	2488	0.31	0.98 (0.85–1.12)	0.86 (0.69–1.08)	0.27
	rs17695623	1744	2901	0.07	0.97 (0.81–1.16)	1.14 (0.52–2.46)	0.86
	rs17161747	1771	2909	0.5	1.13 (0.93–1.38)	1.29 (0.57–2.93)	0.18
	rs17623382	1764	2900	0.12	1.01 (0.87–1.17)	1.01 (0.61–1.66)	0.9
	rs6944385	1758	2893	0.14	1.14 (0.99–1.32)	0.99 (0.66–1.50)	0.14
	rs1267622, rs6944385; AA	1786	2948	0.76	1.02 (0.79–1.33)	1.04 (0.80–1.34)	0.77
*ERBB2*	rs2952155^d^	1667	2678	0.24	1.01 (0.89–1.15)	1.11 (0.84–1.47)	0.57
	rs2952156	1766	2912	0.29	0.97 (0.86–1.10)	1.15 (0.89–1.49)	0.74
	rs1801200	1766	2916	0.22	1.04 (0.92–1.19)	1.01 (0.77–1.31)	0.64
*KRAS*	rs12305513	1788	2934	0.1	0.87 (0.74–1.03)	0.71 (0.38–1.31)	0.053
	rs12822857	1751	2901	0.47	1.01 (0.88–1.17)	0.94 (0.80–1.12)	0.53
	rs10842508	1776	2935	0.25	0.97 (0.86–1.10)	0.95 (0.73–1.22)	0.57
	rs12579073	1765	2900	0.48	0.97 (0.84–1.12)	0.92 (0.78–1.09)	0.36
	rs10842513	1770	2878	0.09	1.03 (0.87–1.21)	0.93 (0.50–1.74)	0.86
	rs4623993	1748	2892	0.16	0.96 (0.83–1.10)	1.13 (0.77–1.67)	0.85
	rs6487464	1763	2895	0.38	1.04 (0.91–1.18)	0.99 (0.82–1.19)	0.94
	rs10842514	1757	2886	0.44	0.98 (0.86–1.13)	1.08 (0.91–1.29)	0.42
	rs11047917^b^^,^^c^	1476	2456	0.06	0.92 (0.75–1.14)	1.62 (0.57–4.57)	0.71
	rs4623993, rs12579073; TC	1717	2818	0.1	0.96 (0.80–1.15)	0.94 (0.56–1.57)	0.63
	rs12822857, rs10842508; AC	1730	2857	0.23	0.99 (0.87–1.13)	1.04 (0.80–1.36)	0.93
	rs12822857, rs10842514; GT	1715	2806	0.4	1.04 (0.91–1.20)	1.12 (0.94–1.34)	0.23
	rs12822857, rs12579073, rs6487464; GAC	1689	2746	0.39	1.04 (0.89–1.21)	1.06 (0.88–1.29)	0.51
*NMI*	rs394884	1708	2852	0.15	1.01 (0.88–1.17)	1.40 (0.84–2.32)	0.47
	rs11551174^b^^,^^c^^,^^d^	1159	2040	0.06	0.96 (0.76–1.23)	1.23 (0.45–3.38)	0.92
	rs289831	1665	2718	0.13	1.05 (0.91–1.22)	1.08 (0.61–1.89)	0.48
	**rs3771886**	1764	2927	0.41	1.03 (0.90–1.18)	**1.19** (**1.00**–**1.42)**	0.075
	**rs11683487**	1464	2564	0.46	**0.80** (**0.69**–**0.93)**	0.87 (0.71–1.02)	**0.038**
	rs2113509	1776	2944	0.13	1.05 (0.91–1.21)	1.16 (0.68–1.97)	0.42
*PIK3CA*	rs2865084^b^^,^^c^^,^^d^	1164	2046	0.06	1.14 (0.89–1.45)	**0.43** (**0.37**–**0.50)**	0.29
	**rs7621329**	1749	2818	0.16	0.99 (0.86–1.13)	1.23 (0.86–1.77)	0.64
	rs1517586	1739	2908	0.1	0.98 (0.83–1.15)	0.77 (0.42–1.40)	0.54
	rs2699905	1741	2855	0.27	1.01 (0.88–1.15)	0.89 (0.71–1.11)	0.49
	rs7641889	1779	2939	0.07	0.89 (0.74–1.07)	1.28 (0.58–2.84)	0.38
	rs7651265	1794	2883	0.1	0.89 (0.76–1.04)	1.58 (0.89–2.80)	0.54
	rs7640662	1765	2916	0.15	1.02 (0.89–1.17)	0.85 (0.57–1.27)	0.86
	rs2677760	1762	2925	0.49	1.01 (0.87–1.16)	1.04 (0.88–1.23)	0.67

CI=confidence interval; MAF=minor allele frequency; OR=odds ratio; SNP=single-nucleotide polymorphism.

a Compared with common homozygote.

b UKOPS is excluded.

c USC is excluded.

d GEOCS is excluded. Bold text indicates positive results, either by *P*-value or CI ranges that do not cross 1.00.

**Table 4:** Genetic associations identified in the *BRAF*, *KRAS*, *ERBB2*, *NM1* and *PIK3CA* after histological subtype stratification

**Gene**	**SNP**	**Controls**	**Cases**	**HetOR**	**HomOR**	***P*_trend_**
*Endometrioid subtype*						
*ERBB2*	rs1801200	2916	263	1.16 (0.88–1.52)	1.71 (1.05–2.76)	0.0389
*PIK3CA*	rs2865084	2039	183	1.60 (1.03–2.50)	0.30 (0.22–0.42)	0.0344
						
*Mucinous subtype*						
*BRAF*	rs10487888	2694	180	1.32 (0.86–2.03)	1.61 (1.03–2.53)	0.0357
	rs1267622	2880	187	0.67 (0.48–0.94)	0.71 (0.35–1.43)	0.0278
	rs17695623	2901	186	0.47 (0.26–0.86)	0.79 (0.10–6.08)	0.0191
*KRAS*	rs12822857	2901	187	0.74 (0.53–1.04)	0.63 (0.41–0.96)	0.0232
	rs6487464	2895	192	0.61 (0.44–0.85)	0.76 (0.50–1.18)	0.0379
	rs10842514	2885	188	1.13 (0.78–1.64)	2.02 (1.35–3.01)	0.0006
*NMI*	rs11683487	2565	154	0.67 (0.47–0.96)	0.62 (0.39–0.99)	0.0269

SNP=single-nucleotide polymorphism.

**Table 5:** Haplotype analysis results for *BRAF*, *ERBB2*, *KRAS*, *NMI* and *PIK3CA*

**Gene**	**Haplotype**	**Frequency in controls (%)**	**OR (95% CI)**	***P*-value^a^**	**Global *P*-value**
*BRAF*	h13142341	21.5	1		**0.005**
	h13122341	19.4	0.88 (0.76–1.01)	0.07	
	h13122331	11.8	0.96 (0.82–1.12)	0.57	
	h33142341	17.3	0.90 (0.78–1.04)	0.15	
	h33142241	5.2	1.08 (0.88–1.31)	0.48	
	**h33342341**	10.3	**0.81** (**0.68**–**0.95)**	**0.012**	
	h33341344	6.8	0.94 (0.78–1.13)	0.49	
	h32342344	6.1	1.15 (0.95–1.39)	0.14	
*ERBB2*	h231	53.6	1		**0.034**
	**h233**	16	**1.17** (**1.02**–**1.34)**	**0.022**	
	h211	6.6	0.99 (0.82–1.20)	0.9	
	**h411**	16.3	**1.19** (**1.03**–**1.37)**	**0.016**	
	h413	6.5	0.84 (0.68–1.05)	0.12	
*KRAS* block 1	h132	52.1	1		0.16
	h112	22.8	1.00 (0.9–1.11)	0.99	
	h114	15.1	1.03 (0.91–1.16)	0.67	
	h314	9.5	0.89 (0.77–1.04)	0.15	
*KRAS* block 2	h122242	30.6	1		0.56
	h122222	3.1	0.89 (0.64–1.23)	0.48	
	h122422	5.4	0.92 (0.73–1.18)	0.52	
	h124422	4.7	0.96 (0.74–1.24)	0.74	
	h142222	5.5	1.01 (0.82–1.25)	0.91	
	h222222	5.4	0.81 (0.65–1.01)	0.06	
	h222242	12.9	1.00 (0.85–1.17)	0.98	
	h222422	11.9	1.03 (0.88–1.19)	0.75	
	h222424	4.3	0.82 (0.64–1.05)	0.11	
	h224422	10.6	0.98 (0.84–1.14)	0.77	
	h242222	3.2	0.94 (0.70–1.27)	0.69	
*NMI*	h23424	45.9	1		0.26
	**h23443**	33.7	**1.11** (**1.003**–**1.22)**	**0.043**	
	h21443	5.7	1.05 (0.84–1.3)	0.67	
	h43223	11.8	1.09 (0.95–1.25)	0.22	
*PIK3CA*	h42432122	48.2	1		0.69
	h42432124	10.2	0.94 (0.81–1.09)	0.39	
	h42412134	14.8	1.01 (0.89–1.14)	0.91	
	h42212124	9.7	0.98 (0.84–1.14)	0.79	
	h44432324	4	1.02 (0.82–1.28)	0.84	
	h44434324	6.6	0.94 (0.79–1.12)	0.5	
	h14432124	3.9	1.20 (0.97–1.48)	0.102	

CI=confidence interval; MAF=minor allele frequency; OR=odds ratio.

In the haplotypes, the numbers correspond to nucleotides: 1=A, 2=C, 3=G, 4=T. SNP order in haplotypes is 5′–3′ of the genes – *BRAF*: rs10487888, rs1733832, rs1267622, rs13241719, rs17695623, rs17161747, rs17623382 and rs6944385; *ERBB2*: rs2952155, rs2952156 and rs1801200; *KRAS* (block 1): rs12305513, rs12822857 and rs10842508; *KRAS* (block 2): rs12579073, rs10842513, rs4623993, rs6487464, rs10842514 and rs11047917; *NMI*: rs394884, rs11551174, rs289831, rs3771886 and rs11683487; *PIK3CA*: rs2865084, rs7621329, rs1517586, rs2699905, rs7641889, rs7651265, rs7640662 and rs2677760.

a Compared with common haplotype. Bold text indicates positive results, either by *P*-value or CI ranges that do not cross 1.00.
