# Supplementary material for: Tagging single-nucleotide polymorphisms in candidate oncogenes and susceptibility to ovarian cancer
Source: Br J Cancer. 2009 Feb 24;100(6):993–1001. doi: 10.1038/sj.bjc.6604947 (PMC2661781; doi:10.1038/sj.bjc.6604947)
Supplement: Supplementary Tables 1 and 2 [file 6604947x1.doc]

**Supplementary Table 1**: Genotype distributions of tagging SNPs in *BRAF*, *ERBB2*, *KRAS*, *NMI* and *PIK3CA*

| **Gene** | **SNP** | **Controls** | | | | | **Cases** | | | | |
| --- | --- | --- | --- | --- | --- | --- | --- | --- | --- | --- | --- |
|  |  | **AA** | **Aa** | **aa** | **Total** | **MAF** | **AA** | **Aa** | **aa** | **Total** | **MAF** |
| *BRAF* | rs10487888 | 612 | 1296 | 786 | 2694 | 0.47 | 365 | 839 | 476 | 1680 | 0.45 |
|  | rs1733832 | 1801 | 238 | 4 | 2043 | 0.06 | 1013 | 139 | 7 | 1159 | 0.07 |
|  | rs1267622 | 1673 | 1032 | 175 | 2880 | 0.24 | 1022 | 623 | 106 | 1751 | 0.24 |
|  | rs13241719 | 1177 | 1050 | 261 | 2488 | 0.31 | 774 | 679 | 149 | 1602 | 0.31 |
|  | rs17695623 | 2507 | 379 | 15 | 2901 | 0.07 | 1515 | 217 | 12 | 1744 | 0.07 |
|  | rs17161747 | 2611 | 285 | 13 | 2909 | 0.05 | 1575 | 185 | 11 | 1771 | 0.06 |
|  | rs17623382 | 2251 | 604 | 45 | 2900 | 0.12 | 1362 | 376 | 26 | 1764 | 0.12 |
|  | rs6944385 | 2169 | 660 | 64 | 2893 | 0.14 | 1281 | 438 | 39 | 1758 | 0.15 |
| *ERBB2* | rs2952155 | 1541 | 1004 | 133 | 2678 | 0.26 | 944 | 627 | 96 | 1667 | 0.25 |
|  | rs2952156 | 1364 | 1374 | 174 | 2912 | 0.3 | 826 | 822 | 118 | 1766 | 0.3 |
|  | rs1801200 | 1753 | 992 | 171 | 2916 | 0.23 | 1055 | 613 | 98 | 1766 | 0.23 |
| *KRAS* | rs12305513 | 2383 | 520 | 31 | 2934 | 0.1 | 1486 | 286 | 16 | 1788 | 0.09 |
|  | rs12822857 | 810 | 1402 | 689 | 2901 | 0.48 | 507 | 852 | 392 | 1751 | 0.48 |
|  | rs10842508 | 1668 | 1086 | 181 | 2935 | 0.25 | 1025 | 643 | 108 | 1776 | 0.24 |
|  | rs12579073 | 780 | 1424 | 696 | 2900 | 0.49 | 497 | 863 | 405 | 1765 | 0.48 |
|  | rs10842513 | 2384 | 465 | 29 | 2878 | 0.09 | 1468 | 286 | 16 | 1770 | 0.09 |
|  | rs4623993 | 2060 | 765 | 67 | 2892 | 0.16 | 1261 | 441 | 46 | 1748 | 0.16 |
|  | rs6487464 | 1102 | 1333 | 460 | 2895 | 0.39 | 676 | 817 | 270 | 1763 | 0.39 |
|  | rs10842514 | 918 | 1399 | 569 | 2886 | 0.44 | 546 | 829 | 382 | 1757 | 0.45 |
|  | rs11047917 | 2158 | 290 | 8 | 2456 | 0.06 | 1310 | 159 | 7 | 1476 | 0.06 |
| *NMI* | rs394884 | 2120 | 698 | 34 | 2852 | 0.13 | 1250 | 428 | 30 | 1708 | 0.14 |
|  | rs11551174 | 1810 | 221 | 9 | 2040 | 0.06 | 1033 | 119 | 7 | 1159 | 0.06 |
|  | rs289831 | 2106 | 579 | 33 | 2718 | 0.11 | 1263 | 381 | 21 | 1665 | 0.11 |
|  | rs3771886 | 1013 | 1409 | 505 | 2927 | 0.41 | 591 | 833 | 340 | 1764 | 0.43 |
|  | rs11683487 | 754 | 1248 | 562 | 2564 | 0.46 | 494 | 654 | 316 | 1464 | 0.44 |
|  | rs2113509 | 2273 | 636 | 35 | 2944 | 0.12 | 1348 | 404 | 24 | 1776 | 0.13 |
| *PIK3CA* | rs2865084 | 1841 | 198 | 0 | 2039 | 0.05 | 1033 | 127 | 0 | 1160 | 0.06 |
|  | rs7621329 | 1966 | 779 | 73 | 2818 | 0.16 | 1212 | 482 | 55 | 1749 | 0.17 |
|  | rs1517586 | 2375 | 497 | 36 | 2908 | 0.1 | 1432 | 291 | 16 | 1739 | 0.09 |
|  | rs2699905 | 1611 | 979 | 265 | 2855 | 0.26 | 992 | 603 | 146 | 1741 | 0.26 |
|  | rs7641889 | 2557 | 367 | 15 | 2939 | 0.07 | 1563 | 205 | 11 | 1779 | 0.06 |
|  | rs7651265 | 2286 | 571 | 26 | 2883 | 0.11 | 1407 | 318 | 24 | 1749 | 0.11 |
|  | rs7640662 | 2107 | 735 | 74 | 2916 | 0.15 | 1271 | 456 | 38 | 1765 | 0.15 |
|  | rs2677760 | 775 | 1462 | 688 | 2925 | 0.48 | 468 | 871 | 423 | 1762 | 0.49 |

AA- common homozygote; Aa – heterozygote; aa – rare homozygote.

**Supplementary Table 2**: Genotype distributions of *NMI* rs11683487 per study

| **Study** | **Controls** | | | | **Cases** | | | | **MAF*** | **HWE^+^** |
| --- | --- | --- | --- | --- | --- | --- | --- | --- | --- | --- |
|  | **AA** | **Aa** | **aa** | **Total** | **AA** | **Aa** | **aa** | **Total** |  |  |
| SEARCH | 165 | 321 | 138 | 624 | 165 | 204 | 124 | 493 | 0.48 | 0.16 |
| MALOVA | 356 | 549 | 257 | 1162 | 141 | 192 | 83 | 416 | 0.46 | 0.1 |
| GEOCS | 130 | 185 | 98 | 413 | 110 | 138 | 71 | 319 | 0.46 | 0.3 |
| USC(A) | 78 | 96 | 44 | 218 | 66 | 87 | 41 | 194 | 0.41 | 0.26 |
| UKOP | 45 | 112 | 40 | 197 | 32 | 47 | 12 | 91 | 0.46 | 0.12 |
| HOPE | 195 | 295 | 141 | 631 | 96 | 134 | 35 | 265 | 0.46 | 0.26 |
| DOVE | 225 | 343 | 140 | 708 | 152 | 250 | 116 | 518 | 0.44 | 0.64 |
| USC (B) | 124 | 169 | 67 | 360 | 78 | 114 | 45 | 237 | 0.42 | 0.48 |

AA- common homozygote; Aa – heterozygote; aa – rare homozygote. *MAF: minor allele frequency; ^+^ HWE: Hardy-Weinberg equilibrium.
